# Haplotype-sharing analysis using Mantel statistics for combined genetic effects

**DOI:** 10.1186/1471-2156-6-S1-S70

**Published:** 2005-12-30

**Authors:** Lars Beckmann, Christine Fischer, Markus Obreiter, Michael Rabes, Jenny Chang-Claude

**Affiliations:** 1 German Cancer Research Center DKFZ, Heidelberg, Germany; 2 Institute of Human Genetics, University of Heidelberg, Heidelberg, Germany

## Abstract

We applied a new approach based on Mantel statistics to analyze the Genetic Analysis Workshop 14 simulated data with prior knowledge of the answers. The method was developed in order to improve the power of a haplotype sharing analysis for gene mapping in complex disease. The new statistic correlates genetic similarity and phenotypic similarity across pairs of haplotypes from case-control studies. The genetic similarity is measured as the shared length between haplotype pairs around a genetic marker. The phenotypic similarity is measured as the mean corrected cross-product based on the respective phenotypes. Cases with phenotype P1 and unrelated controls were drawn from the population of Danacaa. Power to detect main effects was compared to the *X*^2^-test for association based on 3-marker haplotypes and a global permutation test for haplotype association to test for main effects. Power to detect gene × gene interaction was compared to unconditional logistic regression. The results suggest that the Mantel statistics might be more powerful than alternative tests.

## Background

Recently we proposed a flexible approach to gene mapping of complex diseases, whereby we combine Mantel statistics for space-time clustering with genetic information obtained from haplotypes [1]. It has been shown that haplotype sharing methods are well suited for mapping such genes [2-5]. Mantel statistics were introduced in 1967 to correlate temporal and spatial distributions of cancer, notably childhood leukemia, in a generalized regression approach [6]. The Mantel statistic *M *is the sum of the cross product of the spatial similarity *X*_*ij *_multiplied by the temporal similarity *Y*_*ij *_across all pairs of cases *i *and *j*:



The idea behind this approach is that in the presence of space-time clustering the values of spatial similarity *X*_*ij *_correspond to the values of temporal similarity *Y*_*ij *_for correlated cases *i *and *j*.

## Methods

### Mantel statistics using haplotypes

Here we apply the general approach of Mantel's statistics for space-time clustering (Equation 1) to correlate genetic and phenotypic similarity, and to test for gene × gene interaction. The first statistic has the form:



where *x *denotes a genetic marker, and *i *and *j *are haplotypes. *L*_*ij*_(*x*) denotes the genetic similarity between the haplotypes *i *and *j *at *x*, and is defined as the number of intervals surrounding x that are flanked by markers with the same alleles, i.e., that are identical by state (IBS). The phenotypic similarity for two haplotype copies *i *and *j *derived from individuals *s*_*i *_and *s*_*j *_is defined as the mean corrected product *Y*_*sisj *_= (*y*_*si *_- *μ*)(*y*_*si *_- *μ*), where *y*_*si *_and y_*sj *_are the phenotypes of *s*_*i *_and *s*_*j*_, and *μ *denotes the expectation of the phenotype. Here, we chose *μ *as the sample mean, i.e., *μ *= 0.5. Concordant pairs of affected and concordant pairs of unaffected individuals have the weights *Y*_*sisj *_= 0.25, while discordant pairs have the weights *Y*_*sisj *_= -0.25. Alternative measures of phenotypic similarity were discussed in the framework of sib-pair analysis, e.g., the Haseman-Elston method [7] and the weighted pair-wise correlation statistics [8], as well as in family-based association analysis [9]. The summation is over all pairwise comparisons of haplotypes for *i *≠ *j*, where the haplotypes are derived from case-control studies.

The second statistic is constructed to test for the combined effect of two loci:



The information of the first locus *x *is incorporated as the shared length *L*_*ij*_(*x*). At the second locus only genotype information is used. The variable *z*_*si *_is coded in a dominant way, i.e., *z*_*si *_is 1, if the individual *s*_*i *_carries at least one mutant allele, and 0 otherwise. The measure of genotypic similarity *Z*_*sisj *_is then 1, if *z*_*si *_= *z*_*sj*_, and 0 otherwise.

The summands of the Mantel statistic are highly correlated, and any statistical procedure to test for significance has to take into account the interrelationship of the data. Here, we use a Monte Carlo permutation approach to test for significance, as proposed by Mantel [6]. For *M*_0_(*x*) the phenotype *y*_*si *_is permuted over the individuals. The definition of Z is such that *M*_1_(*x*) is the sum over all comparisons of haplotypes from individuals who have the same genotype coding z at the second locus. To derive the null hypothesis of no statistical interaction, the phenotype *y*_*si *_and the genotype coding *z*_*si *_at the second locus for individual *s*_*i *_are permuted jointly over the individuals, and thus the comparisons of haplotypes derived from discordant individuals are incorporated under the null hypothesis.

### Statistical tests for comparison

#### Main effects

We used two alternative tests for power comparison.

1. We applied the *X*^2^-test for association to 3-marker haplotypes. The region of interest was covered by overlapping sliding windows. The haplotypes consisted of 3 consecutive genetic markers. The test was based on a 2*xk X*^2^-table, with *k-*1 degrees of freedom, where *k *denotes the number of haplotypes that occurred in either the case or the control sample. A *p*-value was assigned to the marker in the center of the window. Note that no tests were performed for the marginal markers.

2. The haplotype assignment software PHASE [10,11] performs a global permutation test for significant differences in haplotype frequencies in case and control groups. PHASE tests the null hypothesis that the case and control haplotypes are a random sample from a single set of haplotype frequencies, versus the alternative that cases are more similar to other cases than to controls. Here, this test was based on 100 permutations due to computational burden.

#### Gene × gene interaction

We compared the test statistic *M*_1_(*x*) using haplotypes to unconditional logistic regression based on the genotypes at 2 genetic markers [12]. The respective genotypes were coded for both the recessive and the dominant model.

### Datasets and genetic data

The case-control study samples for two different samples sizes were drawn from the population Danacaa to limit the analysis to individuals defined by phenotype P1.

In this dataset, two major genes, D1 and D2, interacted in an epistatic model. Mode of inheritance is dominant for both D1 and D2.

Table 1 shows the samples that were used to test for main effects. Major gene D1 is located on chromosome 1. We chose flanking single-nucleotide polymorphisms (SNPs) of the disease locus between C01R0045 and C01R0055 from the initial set of markers (sample A), and additional SNPs and microsatellites from packages 28 and 29 (samples B to D). For major gene D2, which is located at the very end of chromosome 3, we analyzed 6 flanking SNPs C03R0276–0281 (samples E and F). To test for gene × gene interaction, information from both disease loci D1 and D2 were used to define the measures L and Z for *M*_1_(*x*). For samples A-D, the markers in Table 1 were used to define the variable L at gene D1, and the SNPs C03R0276–C03R0281 at gene D2 to define the variable Z. For samples E and F, the markers in Table 1 were used to define the variable L at gene D2, and the SNPs C01R0050–C01R0053 at gene D1 to define the variable Z.

### Software

Haplotype pairs assigned to the unrelated individuals were estimated by the use of the PHASE program [10,11]. PHASE lists the most likely pairs of haplotypes for each individual, together with their posterior probability. The most likely (best) estimate of haplotype pairs was chosen for our analysis. SAS 8.02 (SAS Institute Inc., Cary, NC, USA) was used to test for normality and for logistic regression. All other calculations were performed with software developed within our group. Software for the proposed Mantel statistics is available upon request.

## Results

### Main effects

Table 2 shows the results for the analysis of main effects of genetic markers close to D1 and D2. For D1, the Mantel statistic *M*_0_(*x*) yielded point-wise significant results at the marker position C01R052 (*p *= 0.042), which is the marker closest to D1 for the small sample B. For the large sample D, which included additional SNPs, *M*_0_(*x*) yielded the most significant result at SNP C01R0045 (*p *= 0.014).

*M*_0_(x) did not yield significant results for the markers flanking D2 with small sample size. The most significant SNP in the large sample was C03R0280 (*p *= 0.002). The *X*^2^_hap_-test for association, however, did not produce significant results with either the small or the large samples. The permutation test yielded one globally significant *p*-value of 0.03 in the large sample D.

### Gene × gene interaction

Table 3 shows the results for *M*_1_(*x*). The genetic similarity was defined by the same marker sets as in Table 1. *M*_1_(*x*) yielded significant results for all samples except sample A. The most significant results were at the closest markers for D2 (samples E and F), but not for D1. Logistic regression did not reveal significant results for interaction between SNPs surrounding gene D1 and SNPs flanking D2 for the different samples (results not shown).

## Conclusion

We successfully employed a new approach to map disease predisposing genes in case-control studies based on Mantel statistics that correlate genetic and phenotypic similarity. Two types of gene effects involved in complex diseases were considered: main effects and joint effects.

1. The Mantel statistic *M*_0_(*x*) identified the major gene D2 on chromosome 3 given adequate sample size, whereas the alternative methods failed. Major gene D1 on chromosome 1 was simulated without linkage disequilibrium (LD). LD is necessary for haplotype association methods, therefore *M*_0_(*x*)-as expected-did not map D1 correctly.

We acknowledge that the comparison against the *X*^2 ^association test for 3 marker haplotypes is somewhat unfair, but we know of no other standard association test examining longer haplotypes that is not confronted with problems of huge degrees of freedom and sparse data. Additionally, other more sophisticated haplotype-based methods cannot yet be regarded as standard.

2. The Mantel statistic *M*_1_(*x*) accounted for the joint effects of 2 putative disease loci. Taking the combined effects into account, the results were significant for the major genes D1 and D2 and showed lower *p*-values than the results obtained when considering main effects only.

These results show that main effects might not be detectable if gene × gene interaction is present and not considered in the analysis. Our proposed method *M*_1_(*x*) revealed significant statistical interaction between the genes analyzed in contrast to the results obtained in the logistic regression model.

The proposed Mantel statistics employ haplotypes from case-control data and might not be robust to population stratification. In our analysis, we used samples drawn from the Danacaa population and affection status defined by phenotype P1 to reduce heterogeneity in the data. Population stratification is therefore not a major concern in this analysis. We did not adjust the *p*-values for multiple comparisons in this candidate analysis.

Multiple testing is a serious problem especially if all possible gene × gene interactions increase the multiplicity. We solved the problem in the mean time by implementing a step-down algorithm to take into account multiple testing [13,14].

Comprehensive power comparisons are currently being carried out to reveal under which conditions our approach is more powerful than alternative methods.

## Abbreviations

GAW14: Genetic Analysis Workshop 14

IBS: Identical by state

SNP: Single-nucleotide polymorphism

## Authors' contributions

LB participated in planning, interpreting data, carrying out the statistical analysis, and drafting the manuscript. CF participated in planning, interpreting data, writing the manuscript. MO and MR participated in computation and statistical analysis. JC-C participated in planning, interpreting data, and writing the manuscript. All authors read and approved the final manuscript.

## Figures and Tables

**Table 1:** Study samples used in the analysis

Sample	Number of cases/controls	Replicates used		SNPs/microsatellites	Number of markers
				
		Cases	Controls		
A	200/200	1, 2	3, 4	C01R0045 – 0055	11
B	200/200	1, 2	3, 4	C01R0045 – 0055, D01S0021 – 0024	15
C	400/400	1–4, 18	5–8	C01R0045 – 0046, C01R0050 – 0053, C01R0055	7
D	400/400	1–4, 18	5–8	B01T0555 – 0559, C01R0052, B01T0561 – 0565	11
E	200/200	1, 2	3, 4	C03R0276 – 0281	6
F	400/400	1–4, 18	5–8	C03R0276 – 0281	6

**Table 2:** Results of the Mantel statistic (*x*) and the haplotype-based *X*^2^_hap _– test for main effects

Sample	*M*_0_(*x*)		*X*^2^_hap_		Permutation test global
			
	Nearest marker (*p*-value)	Marker with lowest *p*-value (*p*-value)	Nearest marker	Marker with lowest *p*-value (*p*-value)	
Chromosome 1					
A	C01R0052 (0.778)	C01R0054 (0.273)	C01R0052 (0.648)	C01R0047 (0.159)	0.41
B	C01R0052 (0.042)	C01R0052 (0.042)	(ND^a^)	(ND^a^)	0.09
C	C01R0052 (0.579)	C01R0046 (0.054)	C01R0052 (0.471)	C01R0045 (0.31)	0.81
D	C01R0052 (0.068)	C01R0045 (0.014)	C01R0052 (0.095)	C01R0052 (0.095)	0.03
Chromosome 3					
E	C03R0281 (0.134)	C03R0281 (0.134)	C03R0280^b ^(0.668)	C03R0279 (0.629)	0.68
F	C03R0281 (0.043)	C03R0280 (0.002)	C03R0280^b ^(0.11)	C03R0279 (0.09)	0.15

Presented are *p*-values for the nearest markers of the candidate genes (D1: C01R0052; D2: C03R0281) and the marker with the lowest *p*-value.

^a^X^2^_hap_-test was not performed for data including microsatellites.

^b^No *p*-value is assigned to the marginal genetic markers.

**Table 3:** Results of the Mantel statistic *M*_1_(*x*) to test for gene × gene interaction

Sample, nearest marker	*p*-Value	Marker with lowest *p*-value	*p*-Value
Chromosome 1, C01R0052			0.062
A	0.122	C01R0048	0.007
B	0.064	C01R0048	0.001
C	0.009	C01R0053	0.009
D	0.031	C01R0045	
Chromosome 3, C03R0281			
E	0.02	C03R0281	0.02
F	0.003	C03R0281	0.003

Presented are *p*-values for the nearest markers of the candidate genes (D1: C01R0052, D2: C03R0281) and the marker with the lowest *p*-value.
